# Lumpectomy Patients are at Highest Risk for Opioid Overprescription: A Comparison Between Practice Patterns and OPEN National Guidelines

**DOI:** 10.1245/s10434-024-16823-3

**Published:** 2025-01-12

**Authors:** Emily P. Swafford, Sadhana Anantha, Jenna Davis, Rainya Heath, Allison Draper, Sarah Tevis, Neha Goel, Susan B. Kesmodel, Kristin E. Rojas

**Affiliations:** 1 Vanderbilt University Medical Center, Nashville, TN USA; 2 Miller School of Medicine, University of Miami, Miami, FL USA; 3 Department of Surgery, Broward Health Medical Center, Fort Lauderdale, FL USA; 4 Department of Surgery, University of Colorado, Aurora, CO USA; 5 Sylvester Comprehensive Cancer Center, University of Miami Miller School of Medicine, Miami, FL USA; 6 Division of Surgical Oncology, Dewitt-Daughtry Department of Surgery, University of Miami Miller School of Medicine, Miami, FL USA

## Abstract

**Background:**

Nearly 25% of opioid-related deaths are from prescribed opioids, and the exacerbation of the opioid epidemic by the coronavirus disease 2019 (COVID-19) pandemic underscores the urgent need to address superfluous prescribing. Therefore, we sought to align local opioid prescribing practices with national guidelines in postoperative non-metastatic breast cancer patients.

**Methods:**

A single-institution analysis included non-metastatic breast surgery patients treated between April 2020 and July 2021. ‘Overprescription’ was defined as a discharge prescription quantity of oral morphine equivalents (OMEs) greater than the upper limit of the procedure-specific Michigan Opioid Prescribing Engagement Network (OPEN) recommendations. Univariable and multivariate analyses identified risk factors associated with opioid prescribing.

**Results:**

Overall, 464 patients met the inclusion criteria: 280 patients underwent lumpectomy, and 184 patients underwent mastectomy. 52% of patients were overprescribed opioids, including 74% of lumpectomy patients (*p *< 0.001) and 90% of patients undergoing lumpectomy with axillary surgery (*p *< 0.001). Mastectomy patients were overprescribed less frequently (< 25%). The quantity of opioids prescribed at discharge did not correlate to inpatient opioid requirements (*r* = 0.024, *p *= 0.604). Increased age, tobacco use, and long surgery duration were associated with higher quantities of opioids prescribed at discharge.

**Conclusion:**

Patients undergoing less aggressive breast surgery are at very high risk of overprescription, and real-life prescribing patterns do not correlate to national guidelines or inpatient need. Future work will optimize adherence to procedure-specific guidelines and implement tailored discharge protocols.

Despite increased awareness, the opioid epidemic has worsened in recent years. In 2021, the Centers for Disease Control and Prevention (CDC) estimated that opioid overdose-related deaths reached a record high and surpassed 100,000 annually, a 30% increase from the previous year.^1^ A large proportion of the opioids involved in these deaths stem from physician prescriptions.^2–6^ Postoperative opioid prescriptions are a significant source of excess opioids in the community;^2–6^ yet, 72% of opioids prescribed in the postoperative setting are unused by patients.^4^ Additionally, 5–10% of patients continue using opioids for 3–6 months after surgery, and almost one-third of adults receiving long-term opioid therapy at pain clinics reported that their initial opioid prescription came from a surgeon.^7–9^

Surgeons juggle the responsibility of minimizing postoperative pain for their patients while addressing the societal imperative to decrease unnecessary deaths from opioids. Given the difficulty of accomplishing both, resources and guidelines have been released to aid appropriate opioid prescribing in the postoperative setting.^10^ The Opioid Prescribing Engagement Network (OPEN) guidelines developed by the University of Michigan provide a cohesive set of postoperative prescription guidelines for common surgical procedures based on regularly updated literature.^11^

To compare local breast surgery prescribing patterns with national benchmarks, postoperative opioid prescriptions were compared with the OPEN guidelines, and factors associated with a high risk of overprescription were identified. Prior work surrounding individualized opioid prescribing practices has been associated with improved opioid-related outcomes;^2–16^ therefore, a secondary objective determined whether inpatient opioid requirements correlated with the quantity of opioids prescribed at discharge.

## Methods

Following approval by the Institutional Review Board, this single-institution, retrospective cohort study included adult patients who presented for breast cancer surgeries between April 2020 and July 2021. Patients with metastatic breast cancer at the time of diagnosis and reoperations (i.e., margin re-excision) were excluded.

Demographics and clinical characteristics were extracted from the clinical care setting via retrospective chart review of electronic medical records, and information of interest was collected and stored using the REDCap (Research Electronic Data Capture) tool, a secure, web-based software platform designed to support data capture for research studies, providing (1) an intuitive interface for validated data capture; (2) audit trails for tracking data manipulation and export procedures; (3) automated export procedures for seamless data downloads to common statistical packages; and (4) procedures for data integration and interoperability with external sources.^17,18^

**Fig. 1:**
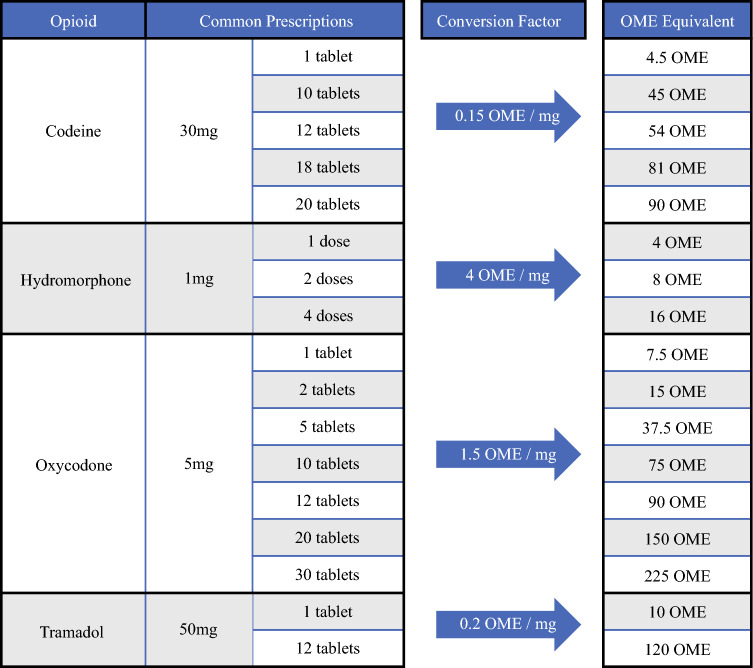
Conversion of common opioid prescriptions to OMEs, utilizing conversion factors. *OMEs* oral morphine equivalents

**Fig. 2:**
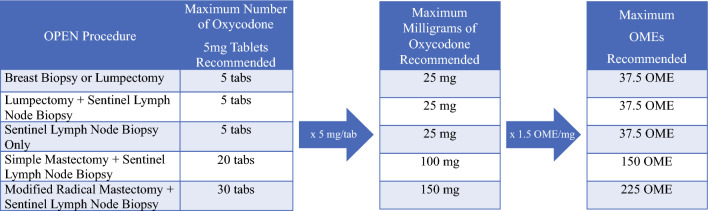
OPEN guidelines with conversion of OPEN recommendations from 5 mg oxycodone tablets to OMEs. *OMEs* oral morphine equivalents, *OPEN* Opioid Prescribing Engagement Network, *tabs* tablets

All opioid medications administered during hospitalization and those prescribed at discharge were quantified via conversion into oral morphine equivalents (OMEs) using conversion factors provided by the CDC opioid equivalence conversion table (Fig. 1).^19^ Opioids administered as part of anesthesia induction were excluded. *Overprescription* was defined as a quantity of prescription OMEs that was greater than the upper limit of the OME range recommended by OPEN guidelines, which was obtained through conversion from 5 mg oxycodone tabs (Fig. 2).

The primary outcomes of interest included the mean difference in discharge OMEs and recommended OMEs, the percentage of patients meeting overprescription criteria, and the mean number of excess OMEs in prescribed and overprescribed subgroups. The correlation between OMEs administered in the hospital and OMEs prescribed at discharge, along with patient factors associated with high opioid requirement and overprescription, were considered secondary outcomes.

**Fig. 3:**
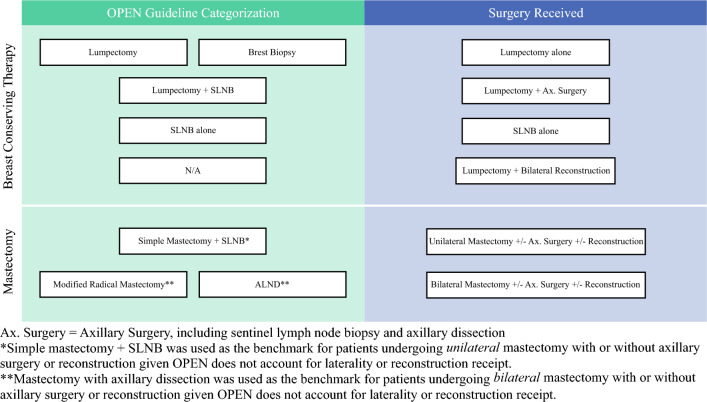
OPEN guideline categorization compared with surgery received

To facilitate comparison, surgical procedures were categorized to best mirror procedure categories utilized in the OPEN guidelines (Fig. 3). Given that the OPEN guidelines lack specificity in terms of laterality and reconstruction receipt, our study placed lumpectomy patients who received sentinel lymph node biopsy (SLNB) and lumpectomy patients who received axillary lymph node dissection (ALND) into one category titled lumpectomy with axillary surgery, utilizing the OPEN recommendations for lumpectomy with SLNB. Additionally, lumpectomy with unilateral reconstruction was included within the appropriate lumpectomy categories (lumpectomy alone or lumpectomy with axillary surgery); however, patients undergoing bilateral mastectomy with and without reconstruction utilized the highest OPEN recommendation for mastectomy patients.

All statistical analysis was performed in IBM SPSS (IBM Corporation, Armonk, NY, USA), with significance set as *p* < 0.05. The univariable analysis identified potential risk factors associated with high inpatient opioid need that were included in subsequent subgroup analyses to assess for significant association. Univariable analysis was used to determine the relationship between inpatient opioid requirements and discharge opioid prescriptions.

## Results

**Fig. 4:**
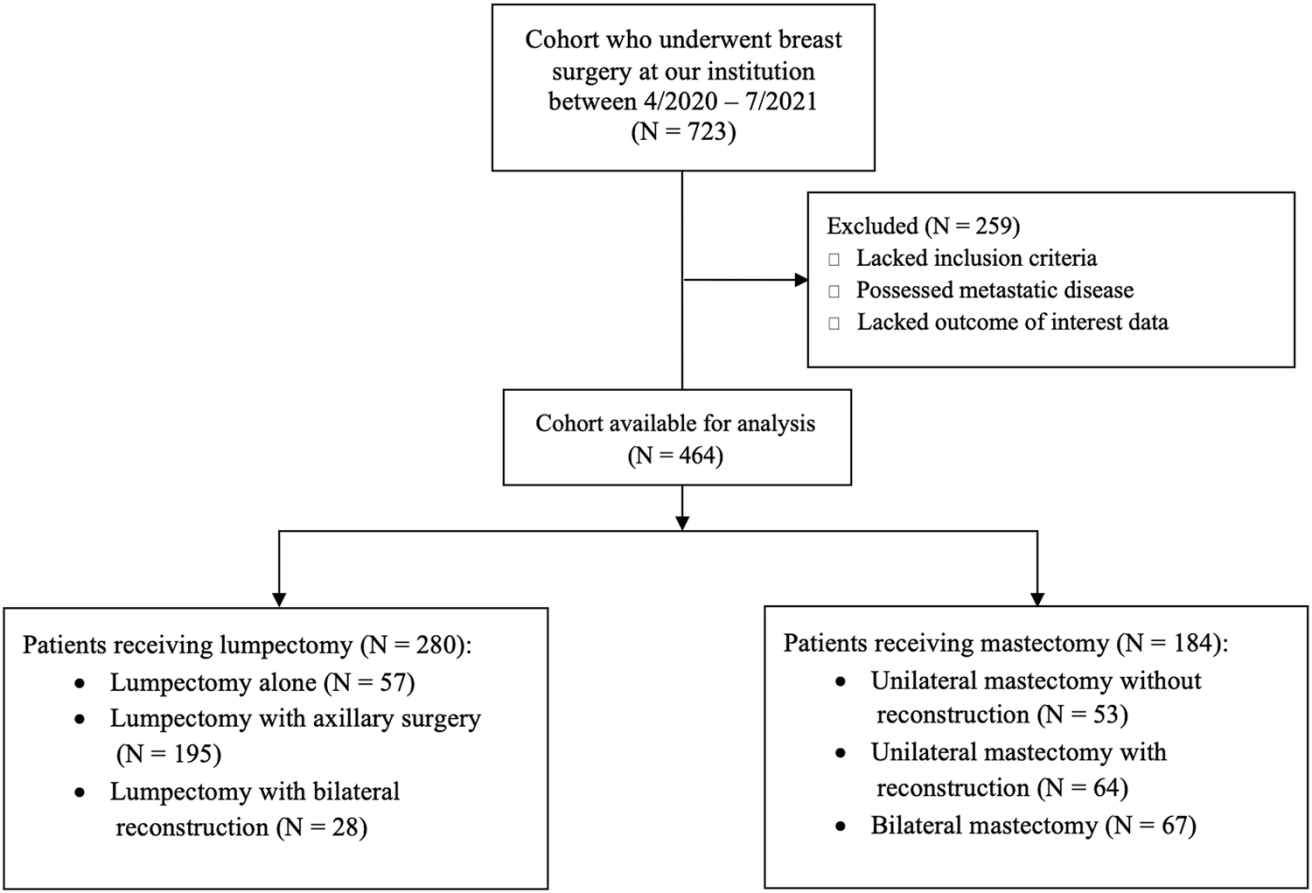
Patient flow diagram

Between April 2020 and July 2021, 723 patients underwent breast surgery, with 464 patients meeting the inclusion criteria, with 259 patients being excluded due to lack of inclusion criteria, possession of exclusion criteria (i.e., presence of metastatic disease), and lack of outcome of interest data (Fig. 4). The mean age of patients was 57 years (standard deviation 12.48). Overall, 376 (81%) patients identified as White, 59 (13%) identified as Black, and 17 (4%) identified as Asian; of the total population, 278 (60%) patients identified as Hispanic. The primary languages spoken included English (58%), Spanish (41%), and Haitian Creole (1%). 280 (60%) patients underwent lumpectomy and 184 (40%) underwent mastectomy. Most patients went home within 24 hours of surgery (Table 1).

**Table 1:** Demographic and clinical characteristics of the study population

	Total study [*N* = 464] *N* (%) or mean (SD)
Age, years	57.85 (12.48)
*Race*	
White	376 (81.0)
Black	59 (12.7)
Asian	17 (3.7)
Unknown	12 (2.6)
*Ethnicity*	
Non-Hispanic	178 (38.4)
Hispanic	278 (59.9)
Unknown	8 (1.7)
*Primary language*	
English	280 (58.0)
Spanish	196 (40.6)
Haitian Creole	3 (0.6)
Other	4 (0.8)
*Median household income*	
< $50,000	38 (8.2)
$50,000–$99,000	403 (86.9)
$100,000–$150,000	14 (3.0)
> $150,000	6 (1.3)
Unknown	3 (0.6)
*BMI*	
< 18.5	8 (1.7)
18.5–24.9	126 (27.2)
25.0–29.9	181 (39.0)
> 30	149 (32.1)
*Tobacco use*	
Yes	142 (30.6)
No	322 (69.4)
*Charlson comorbidity score*	
0	319 (66.0)
1	107 (22.2)
> 1	57 (11.8)
*Oncologic procedure*	
Lumpectomy	280 (60.3)
Mastectomy	184 (39.7)
*Axillary management*	
No axillary surgery	74 (15.9)
SLNB	305 (65.7)
ALND	85 (18.3)
Lymphovenous reconstruction	40 (8.6)
*Breast reconstruction*	
None	317 (68.3)
Unilateral	51 (11.0)
Bilateral	96 (20.7)
Surgical duration, min	166.51 (112.52)
< 120	229 (49.4)
121–240	125 (26.9)
> 241	110 (23.7)
*Length of admission, days*	
0	249 (53.7)
1	197 (42.5)
2	11 (2.4)
>3	7 (1.5)

*SD* standard deviation, *BMI* body mass index, *SLNB* sentinel lymph node biopsy, *ALND* axillary lymph node dissection

Overall, patients received a mean of 13 OMEs during admission and 104 OMEs at discharge postoperatively. 241 (52%) of the patients met the overprescription criteria based on OPEN guidelines for all surgical modalities. Using our predefined criteria, 78% of patients undergoing any type of breast conservation were overprescribed. 74% of patients undergoing lumpectomy alone and 90% of those undergoing lumpectomy with axillary surgery were overprescribed, with a mean of 36 and 50 OMEs, respectively, over the maximum recommended amount. In contrast, only 14% and 25% of those undergoing mastectomy with reconstruction were overprescribed (Table 2).

**Table 2:** Opioids prescribed at discharge compared with the OPEN guidelines

Procedure	*N* (%)	Patients overprescribed [*n* (%)]	OPEN guideline maximum (OMEs)	Mean difference (prescribed vs. recommended OMEs)	*p*-Value
Lumpectomy alone	57 (12)	42 (74)	37.5	+ 36.4	**< 0.001**
Lumpectomy + ax. surgery	195 (42)	175 (90)	37.5	+ 50.3	**< 0.001**
Lumpectomy + bilateral reconstruction	28 (6)	NA	NA	NA	NA
Unilateral mastectomy ± ax. surgery (no reconstruction)	53 (11)	4 (8)	150	+ 54.4	0.089
Unilateral mastectomy ± ax. surgery with reconstruction	64 (14)	16 (25)	150	+ 62.2	**0.017**
Bilateral mastectomy ± ax. surgery (no reconstruction)	14 (3)	2 (14)	225	+ 33.8	0.070
Bilateral mastectomy ± ax. surgery with reconstruction	53 (11)	2 (4)	225	+ 50.0	0.063

*ax. surgery* axillary surgery, including sentinel lymph node biopsy and axillary dissection, *OPEN* Opioid Prescribing Engagement Network, *OMEs* oral morphine equivalents, *NA* not available

Bolded *p*-values indicate statistical significance

Univariable analysis revealed that high inpatient opioid requirements was associated with older age (*p *= 0.025), more comorbidities (*p *= 0.016), longer surgical duration (*p *= 0.003), and greater length of admission (*p *< 0.001). Age (*p *< 0.001), tobacco use (*p *= 0.003), type of surgical procedure (*p *< 0.001), and surgical duration (*p *< 0.001) was associated with higher amounts of OMEs prescribed at discharge. Race, ethnicity, primary language, median household income, body mass index (BMI), and Charlson comorbidity score were not significantly associated (Table 3). After correcting for age, tobacco use, and surgery time, inpatient and discharge OMEs were not positively correlated (*r* = 0.024, *p* = 0.604), which signifies that inpatient opioid need does not appear to impact the quantity of opioids in the discharge prescription.

**Table 3:** Patient factors associated with greater inpatient opioid need and discharge opioid amount

	Total study [*N* = 464]	Inpatient OMEs	*p* value	Discharge OMEs	*p* value
*Age, years*					
<40	29 (6.3)	31.62 (66.75)	**0.025**	131.57 (61.13)	**<0.001**
40–64	300 (64.7)	12.24 (39.90)		108.87 (58.70)	
>65	135 (29.0)	9.92 (28.41)		86.12 (65.81)	
*Race*					
White	376 (81.0)	10.62 (28.32)	0.112	105.56 (61.66)	0.409
Black	59 (12.7)	22.70 (79.41)		96.16 (66.62)	
Asian	17 (3.7)	19.64 (29.70)		104.29 (66.62)	
Unknown	12 (2.6)	21.75 (52.60)		80.38 (45.16)	
*Ethnicity*					
Non-Hispanic	178 (38.4)	15.55 (50.26)	0.482	99.64 (65.72)	0.381
Hispanic	278 (59.9)	11.13 (31.26)		106.70 (60.09)	
Unknown	8 (1.7)	8.44 (7.17)		87.75 (46.83)	
*Primary language*					
English	280 (58.0)	13.78 (42.51)	0.825	101.46 (65.07)	0.564
Spanish	196 (40.6)	11.81 (35.54)		107.22 (57.99)	
Haitian Creole	3 (0.6)	1.50 (2.60)		83.00 (76.27)	
Other	4 (0.8)	0.70 (1.40)		76.50 (56.21)	
*Median household income*					
<$50,000	38 (8.2)	23.78 (64.96)	0.242	117.42 (57.96)	0.611
$50,000–$99,000	403 (86.9)	11.76 (36.94)		102.88 (63.13)	
$100,000–$150,000	14 (3.0)	7.71 (12.50)		91.39 (41.71)	
>$150,000	6 (1.3)	7.25 (15.88)		106.50 (48.66)	
Unknown	3 (0.6)	44.17 (43.52)		87.00 (90.15)	
*BMI*					
<18.5	8 (1.7)	7.81 (14.75)	0.238	91.13 (55.50)	0.896
18.5–24.9	126 (27.2)	12.39 (40.94)		102.95 (64.59)	
25.0–29.9	181 (39.0)	9.07 (17.22)		105.74 (66.27)	
>30	149 (32.1)	17.88 (55.11)		102.42 (55.24)	
*Tobacco use*					
Yes	142 (30.6)	10.62 (28.00)	0.165	97.28 (56.49)	**0.003**
No	322 (69.4)	17.68 (57.35)		117.61 (71.75)	
*Charlson comorbidity score*					
0	319 (66.0)	9.79 (17.98)	**0.016**	104.24 (55.89)	0.851
1	107 (22.2)	14.31 (53.19)		100.45 (79.93)	
>1	57 (11.8)	25.93 (75.71)		104.97 (58.49)	
*Oncologic procedure*					
Lumpectomy	280 (60.3)	7.91 (35.20)	0.304	80.16 (54.27)	**< 0.001**
Mastectomy	184 (39.7)	20.19 (44.23)	0.202	139.44 (56.09)	**< 0.001**
*Axillary management*					
No axillary surgery	74 (15.9)	8.49 (25.68)	0.081	74.00 (73.63)	**< 0.001**
SLNB	305 (65.7)	11.48 (38.72)		103.26 (58.27)	
ALND	85 (18.3)	21.15 (49.83)		130.01 (54.95)	
Lymphovenous reconstruction	40 (8.6)	19.51 (34.61)	0.211	133.57 (56.06)	**< 0.001**
*Breast reconstruction*					
None	317 (68.3)	9.87 (40.64)	0.066	81.96 (47.64)	**< 0.001**
Unilateral	51 (11.0)	18.57 (30.46)		151.89 (75.50)	
Bilateral	96 (20.7)	19.29 (38.89)		148.41 (58.61)	
*Surgical time, min*	166.51 (112.52)				
<120	229 (49.4)	6.80 (35.82)	**0.003**	73.32 (40.51)	**< 0.001**
121–240	125 (26.9)	15.77 (41.50)		114.88 (57.92)	
>241	110 (23.7)	21.82 (42.40)		152.91(67.98)	
*Length of admission, days*	0.52 (0.63)				
0	249 (53.7)	6.25 (35.99)	**< 0.001**	76.54 (47.12)	**< 0.001**
1	197 (42.5)	18.54 (42.37)		133.90 (62.99)	
2	11 (2.4)	37.64 (29.80)		142.71 (80.35)	
>3	7 (1.5)	7.86 (1.95)		43.73 (36.08)	

Data are expressed as *n* (%) or mean (SD)

Bolded *p*-values indicate statistical significance

*BMI* body mass index, *SLNB* sentinel lymph node biopsy, *ALND* axillary lymph node dissection, *OMEs* oral morphine equivalents, *SD* standard deviation

## Discussion

Despite heightened attention to the opioid epidemic, postoperative opioid prescribing persists as a contributing factor to opioid diversion and misuse. Our study demonstrates that opioid prescriptions after breast cancer surgery oftentimes exceed OPEN guidelines and that patients undergoing breast-conserving surgery with lumpectomy are at the highest risk. Factors such as increasing age, tobacco use, and longer surgery duration were significantly associated with increased OMEs on discharge prescriptions in our study population, while inpatient opioid need did not appear to influence the discharge opioid prescription.

Our study aligns with findings from other research and reveals substantial deviation in postoperative OME prescriptions, underscoring the persistence of widely variable opioid prescribing patterns among surgeons.^12,13,20,21^ Institutional factors such as trainee prescribers, provider preference and biases, and lack of education on available guidelines and appropriate prescribing factors likely play a role. Specifically, at academic institutions, such as the institution utilized in this study, prescribers differ in many ways, including by individual, specialty (i.e. breast surgery vs. surgical oncology), and level (i.e. attending vs. fellow vs. resident). Individual surgeon preference when estimating a patient’s opioid needs plays a role in prescription variability and has been shown by Park et al. to be misaligned with patient-reported use.^21^ Future work studying the variability in prescribing practices by specialty and level may provide further insight; however, current data surrounding opioid prescribing practices between specialty and level remains mixed.^22^

Separately, the observed variability may be linked to a lack of awareness regarding standardized recommendations, such as the OPEN guidelines, and broader dissemination efforts could enhance adherence. In an era of widespread multimodal analgesia practices, improved provider education on actual opioid requirements for patients undergoing less aggressive surgery should be developed to mitigate superfluous opioid prescribing and opioid diversion.

Moreover, while this study collected data on enhanced recovery after surgery (ERAS) protocols, very few providers at our institution utilized these methods. Providers who utilized these protocols generally prescribed fewer opioids. Furthermore, patients undergoing larger surgeries (i.e. mastectomy, reconstruction, etc.) were more likely to undergo an ERAS protocol, which perhaps contributed to their decreased risk of overprescription compared with those undergoing smaller surgeries (i.e. lumpectomy with or without axillary surgery).

Additionally, while the OPEN guidelines do provide a set of postoperative prescription recommendations, our work demonstrates their lack of granularity, as both laterality and reconstructive options in breast surgery were notably absent.^11^ Immediate breast reconstruction has been associated with improved aesthetic outcomes and has increased in popularity in recent years.^23,24^ However, despite the frequency of these procedures, the OPEN guidelines do not provide opioid prescribing recommendations for breast surgeries that involve simultaneous reconstruction.

Tailored opioid prescribing patterns, such as using inpatient opioid requirement as a benchmark for discharge prescriptions, have been associated with opioid-related benefits such as reduced unused opioids, lower refill rates, and decreased overall opioid consumption.^12,25–28^ Given the lack of correlation between inpatient opioid use and discharge prescriptions, there remains significant room for improvement in personalized opioid prescribing. Strategies that use inpatient opioid needs to better estimate discharge prescriptions may be underexplored and could enhance individualization, ultimately leading to improved opioid-related outcomes.

Limitations to this study include its retrospective nature and location at an academic, tertiary hospital, which may prevent the results from being broadly generalizable. Second, opioids taken could not be distinguished from opioids prescribed in the outpatient setting for this study; however, this information remains challenging to collect given it is often subject to reporting bias.^29^ Preoperative opioid use is documented from the medication list in our study but may not account for prescriptions from outside institutions and is, therefore, unable to be incorporated as a risk factor for higher opioid prescribing at discharge. Additionally, our analysis may have been negatively impacted by the lack of OPEN guideline specificity for laterality and reconstruction during our surgical procedure categorization, suggesting that more granular guidelines are necessary given the lack of recommendations for reconstructive procedures, which are becoming common.^24^

Our findings highlight the need for improved guideline awareness and adherence as well as prescription individualization. Future studies utilizing tailored discharge prescription protocols should quantify post-discharge outcomes to better understand their role in improving opioid prescribing practices postoperatively. Systemic changes, such as electronic medical record prompts illuminating inpatient OME consumption, have the potential to aid in adherence to individualized opioid prescriptions and hence warrant further investigation. Additionally, studies that measure the rate of refill requests as well as actual refill orders could help assess the outcome of appropriate and excessive opioid prescriptions.

## Conclusion

Continued evaluation of current opioid prescribing patterns is the critical first step in limiting opioid overprescription and improving the tenacious opioid epidemic. The present study demonstrates that opioid prescribing practices following breast cancer surgery still significantly exceed recommended guidelines, especially in patients undergoing ‘less aggressive’ surgery, and are not reflective of individualized inpatient opioid requirements. Future work will seek to improve adherence to procedure-specific guidelines and to implement a tailored discharge prescription protocol to decrease the quantity of opioids available for diversion in our communities.
