# Correction: Lumpectomy Patients are at Highest Risk for Opioid Overprescription: A Comparison Between Practice Patterns and OPEN National Guidelines

**DOI:** 10.1245/s10434-025-17010-8

**Published:** 2025-02-07

**Authors:** Emily P. Swafford, Sadhana Anantha, Jenna Davis, Rainya Heath, Allison Draper, Sarah Tevis, Neha Goel, Susan B. Kesmodel, Kristin E. Rojas

**Affiliations:** 1 Vanderbilt University Medical Center, Nashville, TN USA; 2 Miller School of Medicine, University of Miami, Miami, FL USA; 3 Department of Surgery, Broward Health Medical Center, Fort Lauderdale, FL USA; 4 Department of Surgery, University of Colorado, Aurora, CO USA; 5 Sylvester Comprehensive Cancer Center, University of Miami Miller School of Medicine, Miami, FL USA; 6 Division of Surgical Oncology, Dewitt-Daughtry Department of Surgery, University of Miami Miller School of Medicine, Miami, FL USA

**Correction to: Annals of Surgical Oncology** 10.1245/s10434-024-16823-3

The following author disclosures are missing from the original article:

Dr. Rojas received speaker honoraria from Pacira Pharmaceuticals in 2023. Dr. Goel received support from the Breast Cancer Research Foundation, V Foundation Grant Award, American Society of Clinical Oncology (ASCO) Career Development Award, and American Surgical Association Fellowship Award. All other authors have nothing to disclose.

